# Supplementation of zinc-chelating peptides from *Holothuria scabra* improve zinc status in the offspring of zinc-deficient rats

**DOI:** 10.3389/fnut.2025.1693713

**Published:** 2026-01-09

**Authors:** Gita Syahputra, Melva Louisa, Nunik Gustini, Dwi Endah Kusumawati, A’liyatur Rosyidah, Masteria Yunovilsa Putra, Heri Ahmad Sukria, Puspita Eka Wuyung, Fadilah Fadilah, Hee Jae Lee

**Affiliations:** 1 Department of Pharmacy, Faculty of Pharmacy, Universitas Islam Sultan Agung, Semarang, Indonesia; 2 Research Center for Vaccine and Drugs, National Research and Innovation Agency, Jakarta, Indonesia; 3 Department of Pharmacology and Therapeutics, Faculty of Medicine, Universitas Indonesia, Jakarta, Indonesia; 4 Department of Pharmacy, Faculty of Pharmacy, Universitas Indonesia, Depok, Indonesia; 5 Department of Nutrition and Food Technology, Bogor Agricultural University, Bogor, Indonesia; 6 Department of Pathology Anatomy, Faculty of Medicine, University of Indonesia, Jakarta, Indonesia; 7 Animal Research Facility, Indonesia Medical Education and Research Institute, Jakarta, Indonesia; 8 Department of Medical Chemistry, Faculty of Medicine, University of Indonesia, Jakarta, Indonesia; 9 Department of Pharmacology, School of Medicine, Kangwon National University, Chuncheon, Republic of Korea

**Keywords:** metallothionein, zinc deficiency, zinc transporter, ZIP2, ZIP4, ZnSO_4_

## Abstract

**Introduction:**

Zinc deficiency in children remains a significant health risk, impairing their physical and cognitive maturation. Supplementation with peptide-bound zinc is thought to offer better bioavailability than inorganic zinc salts. *Holothuria scabra*, commonly referred to as a sea cucumber, contains peptides that may chelate zinc, potentially improving its absorption. The present study aimed to evaluate the efficacy of zinc-chelating peptides from *Holothuria scabra* (ZCP) supplementation in rat offspring born to zinc-deficient parental rats.

**Methods:**

Pregnant Sprague Dawley rats were fed either a standard zinc diet (40 mg of zinc per kg of feed) or a zinc-deficient diet (4 mg of zinc per kg of feed). Twelve rat offspring born on the standard zinc diet were randomly assigned to receive either vehicle only or ZCP 40 mg/kg BW. Twenty-four offspring born from zinc-deficient maternal rats were divided into groups that received vehicle only, ZCP 4 mg/kg BW, ZCP 40 mg/kg BW, or ZnSO_4_ 40 mg/kg BW for 3 weeks. At the end of the treatment, serum and intestinal samples were collected and analysed for zinc and metallothionein concentrations, as well as for ZIP2 and ZIP4 mRNA expressions.

**Results:**

ZCP supplementation significantly increased serum and duodenal zinc levels, metallothionein concentration, and mRNA expressions of ZIP2/ZIP4 in zinc-deficient rat offspring compared to untreated zinc-deficient ones. Supplementation with ZCP at 4 mg/kg BW showed superior or equivalent improvements compared with ZnSO_4_ in most parameters.

**Conclusion:**

Zinc chelating peptides from *Holothuria scabra* improved zinc plasma and duodenal concentrations by enhancing the expression of zinc transporters in zinc-deficient rat offspring. ZCP is a promising alternative to conventional inorganic zinc supplements.

## 1 Introduction

Zinc (Zn) is a vital nutrient essential for optimal growth and development during the first 1,000 days of life, encompassing pregnancy and the first 2 years of the child’s life. This period is vital for embryogenesis, fetal development, and infants’ physical and cognitive growth (1, 2). Numerous studies have demonstrated that maternal zinc status directly affects fetal development (3–5). Furthermore, research suggested that there is a substantially high rate of pregnant women with zinc deficiency, with a prevalence rate ranging from 50% to 90% (6).

Despite the lack of population-based estimates of zinc deficiency in infants, it is recognized that mild to moderate zinc deficiency due to inadequate dietary intake is prevalent worldwide. Children aged less than 5 years and those exposed to zinc-deficient diets will benefit from daily supplementation of zinc to reduce the health consequences of zinc deficiency (7–9).

There are several forms of zinc supplementation available, including inorganic zinc, organic zinc, and zinc chelate. Among the three types of zinc supplements, first-generation, inorganic zinc, is the most commonly used (10). However, Zn sulphate has been associated with the onset of gastrointestinal disorders, including nausea and vomiting, mostly at high doses. The second generation of zinc supplements comprises organic forms, including zinc gluconate, zinc oxalate, zinc citrate, and zinc lactate. Second-generation zinc supplements have been shown to reduce the incidence of gastrointestinal disorders compared to first-generation supplements. The absorption rates of first- and second-generation Zn supplements depend on their solubility (10, 11). Third-generation zinc supplements are Zn-chelated with peptide-like molecules. Third-generation Zn supplements are designed to reduce the incidence of adverse effects, enhance the rate and extent of absorption, and improve Zn bioavailability (12–15). Research has demonstrated that Zn chelating peptides (Z) are supplements with high solubility and stability in the gastrointestinal tract (13, 15). Research on third-generation Zn supplements is currently extensive, and as a result, these supplements are not yet widely used as a standard therapy for zinc deficiency (11).

Peptide-like molecules can also be sourced from sea cucumber, a marine organism that comprises 70% collagen fibers, which is valuable as a source of peptides beneficial to health (16–19). *Holothuria scabra* is a type of sea cucumber. In a previous study, we reported the successful extraction of collagen, the identification of the active fraction, and the determination of peptide sequences that exhibit potent zinc-chelating properties (17, 20, 21). Moreover, the active fraction of zinc-chelating peptides (ZCP) has been evaluated for Zn absorption *ex vivo* using the everted gut sac model (21). However, the efficacy of the zinc-chelating peptide from *Holothuria scabra* in an *in vivo* model of zinc deficiency remains unknown. Thus, the present study aimed to investigate the efficacy of ZCP from *Holothuria scabra* in improving zinc status in offspring born to zinc-deficient parental rats.

## 2 Materials and methods

### 2.1 Animals and treatments

This study was approved by the Research Ethics Committee of the Faculty of Medicine, Universitas Indonesia (No. KET-309/UN2.F1/ETIK/PPM/00.00/2022). The treatment was performed on rat offspring born to parental rats fed with a standard diet or a zinc-deficient diet. This investigation employed adult male and female Sprague-Dawley rats, weighing 150–200 grams at 8 weeks of age. The animal subjects were obtained from the Animal Research Facilities at the Faculty of Medicine, University of Indonesia, and maintained in a room with a constant temperature of 22–25°C and constant humidity (45–65% RH). The lighting schedule was kept at an adequate level, with 12 h of light and 12 h of dark. The rats were provided with fed pellet diets and had *ad libitum* access to drinking water. The treatment of the animals followed the Guidelines provided by the Animal Research Facility of Universitas Indonesia (National Accreditation No. LP-1855-IDN).

**FIGURE 1:**
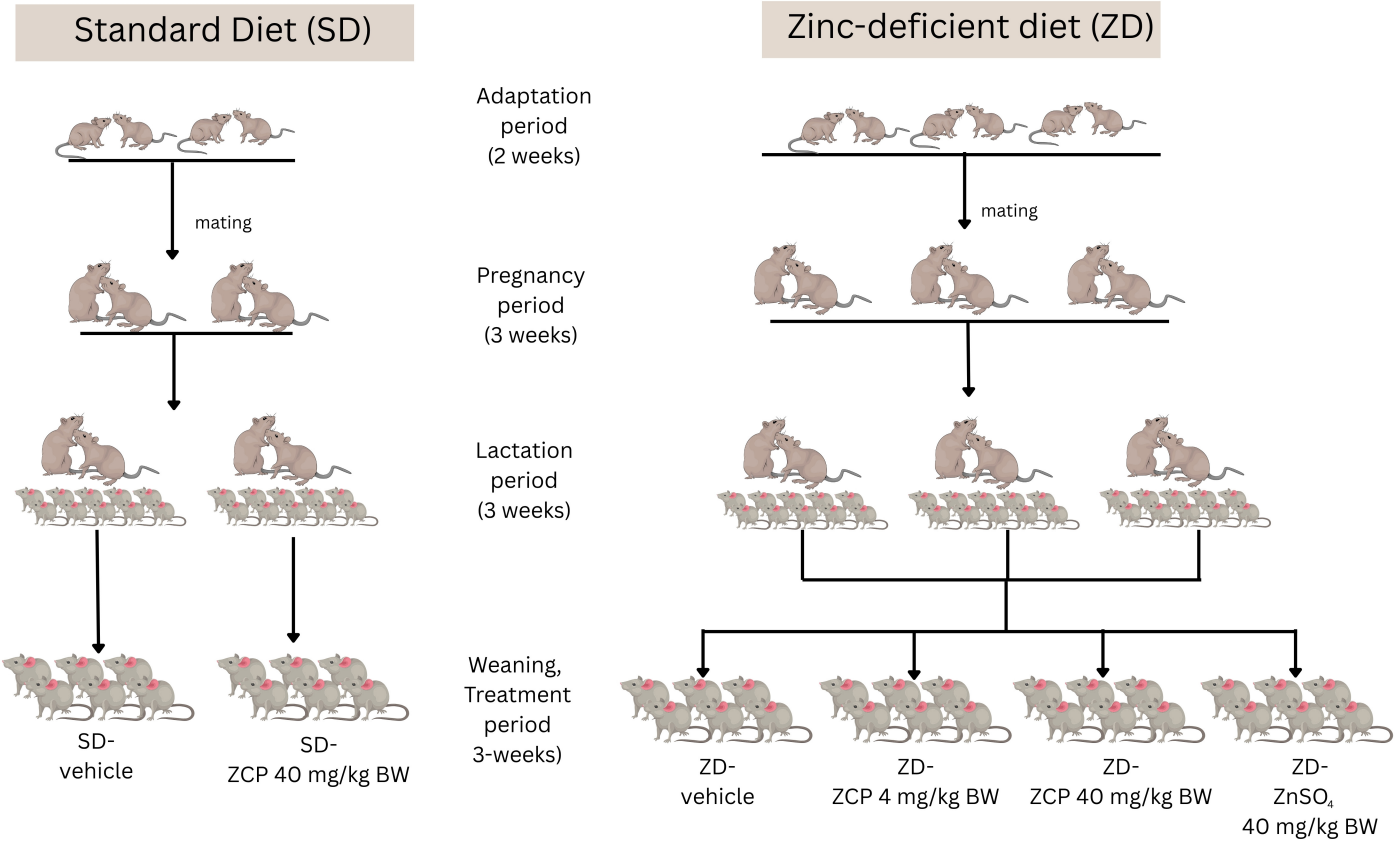
Experimental designs of zinc-chelating peptides from Holothuria scabra (ZCP) in the offspring of maternal zinc deficiency. Male and female rats were fed either a standard diet or a zinc-deficient diet during adaptation, pregnancy, and lactation. The maternal rats remained with the males until weaning. The weanlings received treatments for 3 weeks, which included: standard diet + vehicle (*n* = 6); standard diet + ZCP 40 mg/kg BW; zinc-deficient diet + vehicle; zinc-deficient diet + ZCP 4 mg/kg BW; zinc-deficient diet + ZCP 40 mg/kg BW; zinc-deficient diet + ZnSO_4_ 40 mg/kg BW.

In short, an equal number of male and female rats (*n* = 10) were divided into standard diet or zinc-deficient groups. During the two-week adaptation before the mating period, two pairs of adult rats (*n* = 4) were fed a standard diet, and three pairs of rats (*n* = 6) were fed a low zinc diet. Each rat received the same type of diet throughout gestation and lactation. The maternal rats were kept with the male rats until the end of weaning. All offspring were weaned at 3 weeks (day 21 of lactation). Then, the offspring from each maternal rat fed with standard diets were randomized into two groups of six rats: vehicle only or ZCP 40 mg/kg BW. Offspring from each zinc-deficient maternal rat were also randomized into four groups of six rats: vehicle only, ZCP 4 mg/kg BW, ZCP 40 mg/kg BW, or ZnSO_4_ 40 mg/kg BW. At the end of the study, blood and intestines were collected for further analysis (Figure 1).

The doses of ZCP and ZnSO_4_ used in treatments were calculated based on the amount of pure zinc in its unbound form. Laboratory results showed ZCP contained 92.11 mg of zinc per 100 mg of zinc peptides, while ZnSO_4_ contains 22.74 mg of zinc per 100 mg of its preparation. We then calculated the doses of ZCP and ZnSO_4_ needed to reach 4 mg/kg BW and 40 mg/kg BW for ZCP, and 40 mg/kg BW for ZnSO_4_. Each rat’s body weight was measured prior to supplementation so that the dose administered matched the individual rat’s body weight.

### 2.2 Diet composition

The diet given to the rats differed only in zinc content, with 40 mg of zinc per kg of feed in the standard diet group and 4 mg of zinc per kg of feed in the zinc-deficient group. The other diet composition included 650 g/kg of carbohydrate, 100 g/kg of fat, 200 g/kg of protein, a mineral mix of 42 mg/kg (consisting of Ca, P, Fe, Na, Cl, and Mg), and a vitamin mix of 0.25 mg/kg.

### 2.3 Zinc-chelating peptides from *Holothuria scabra* (ZCP)

The zinc-chelating peptides from *Holothuria scabra* used in the present study have been characterized previously (17, 21). In short, the ZCP consists of six zinc peptides: Asp-Asp-Ala-Phe-Gln-Ala-Phe-Cys; Thr-Asp-Asn-Leu; Leu-Gly-Cys; Pro-Gly-Thr; Ser-Cys; Pro-Tyr, with molecular weights ranging from 208 -915 Da. The peptides exhibited a maximum zinc binding capacity of 92.11 mg/100 g at a molecular weight of 30 kDa (21).

### 2.4 Zinc concentrations

Zinc levels in serum and duodenal homogenate from parental rats and offspring were measured following the protocol detailed in the Zinc Assay Kit (Merck, USA). The quantification was performed according to the protocol, and the samples were measured using a microplate reader at 560 nm.

### 2.5 *Hemoglobin* measurement

The fresh rat blood sample, collected in a tube with EDTA as the anticoagulant, was analyzed for its parameters using a fully automated hematology analyzer (Onetech Medical A) operating at a wavelength of 546 nm.

### 2.6 Metallothionein concentrations

Metallothionein concentrations from the duodenum segments of the offspring were measured using a sandwich enzyme-linked immunoassay kit using Rat-Metallothionein-1 ELISA (Novus Biologicals, USA). The reaction was performed according to the manufacturer’s protocol using a microplate reader at a wavelength of 450 nm.

### 2.7 mRNA expressions of zinc transporters

mRNA expressions of zinc transporters were done on the homogenate of the duodenal segment of the rat offspring. RNA extraction, cDNA synthesis, and quantitative RT-PCR were performed using an Agilent (USA) kit. RNA was extracted from 25 mg of duodenal segment from the small intestines, which were subsequently converted to cDNA.

The mRNA expressions of SLC39A2 and SLC39A4 were analyzed by quantitative RT-PCR on a Bio-Rad CFX96 machine (Bio-Rad, USA) using GAPDH as the housekeeping gene, with primers listed in Table 1. The amount of cDNA template used was 1 ng/1 μL. The PCR master mix solution was prepared for several reactions on the apparatus, with 40 cycles, using the following temperature settings: pre-denaturation at 95 °C for 10 min, denaturation at 95 °C for 15 min, and elongation at 72 °C for 30 s. The annealing temperature used was 60 °C. The data obtained were cycle threshold (Ct) values, which were automatically calculated by the software. The Ct values were then calculated using the Livak method to obtain the mRNA expression levels (22).

**TABLE 1:** Primer sequences used in the study.

Genes	Primer nucleotide sequences	Annealing temperature (^0^Celsius)
GAPDH (F)	GCA AGA GAG AGG CCC TCA G	60
GAPDH (R)	TGT GAG GGA GAT GCT CAG TG	60
SLC39A2 (F)	TTC TCG TGA TGC TGC TGC C	60
SLC39A2 (R)	TAA GTC CCA AGC AAG GAC GG	60
SLC39A4 (F)	CTC GCA ATA TCA CGC TGC CC	60
SLC39A4 (R)	CCT CTG TCA CCA AGT CTG AGC G	60

### 2.8 Statistical analysis

The data analysis was conducted using an unpaired Student’s *t*-test to compare two groups. For the six groups of rats, the analysis was performed with One-Way ANOVA followed by a Tukey *post hoc* test if the data was normally distributed, or the Kruskal-Wallis test followed by the Mann-Whitney U test if not. Data were analyzed after conducting a normality test using the Shapiro-Wilk test. Results are presented as the mean ± SEM, with *p* < 0.05 considered statistically significant at a 95% confidence level. All analyses and plots were generated using Prism 10.6.0 (GraphPad, CA, USA).

## 3 Results

### 3.1 Low serum and duodenum zinc concentrations in the adult rats after zinc-deficient diets

**FIGURE 2:**
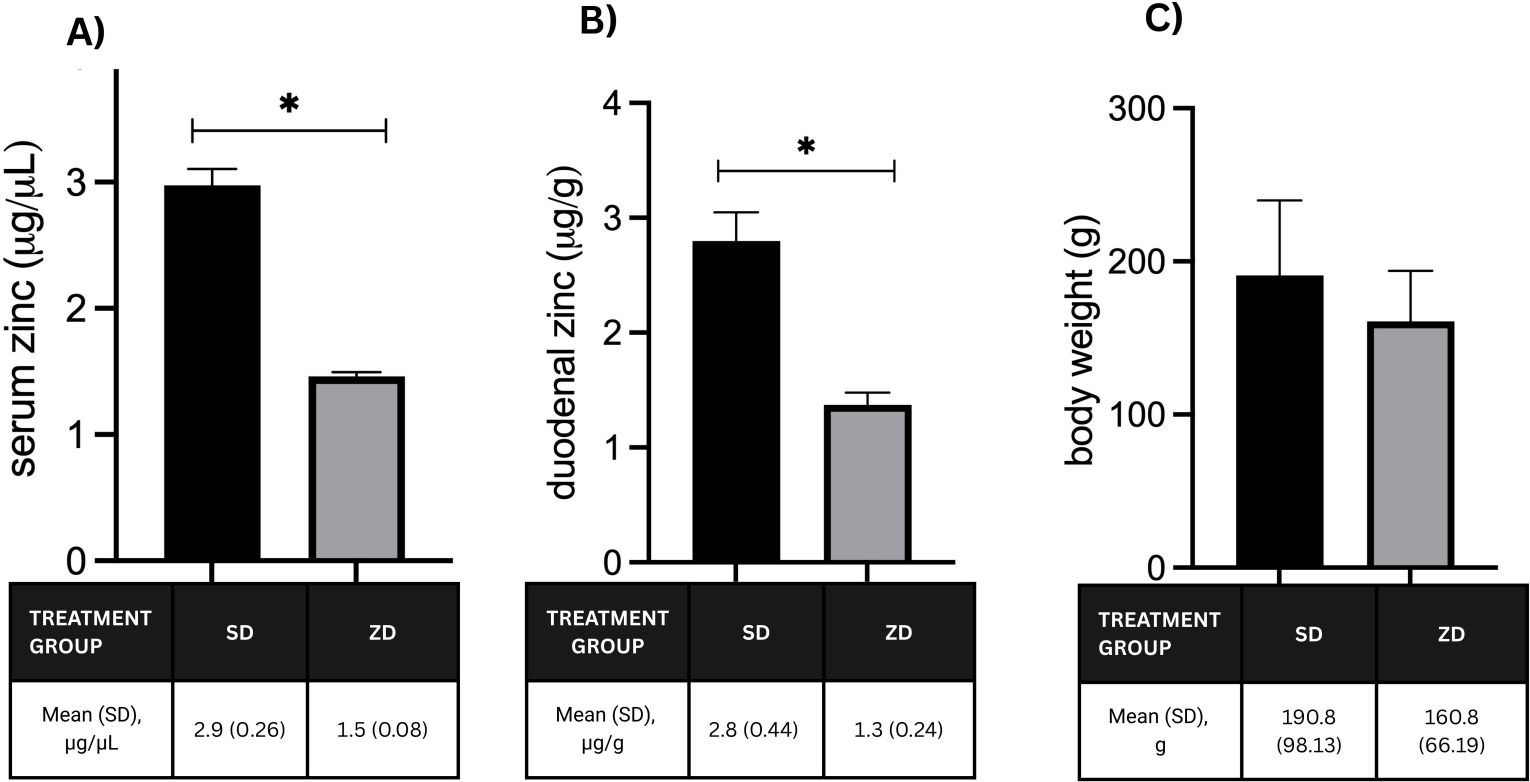
Zinc concentrations in the serum **(A)** and duodenum **(B)** of the parental rats, **(C)** Body weight of the parental rats. SD, standard diet; ZD, zinc-deficient diet; *p < 0.05 vs SD.

We successfully established zinc deficiency in adult rats by administering a low-zinc diet throughout the adaptation, gestation, and lactation phases, as evidenced by significantly lower serum and duodenal zinc concentrations compared with the standard diet group (Figures 2A, B). However, zinc deficiency only resulted in a slight decrease in body weight (Figure 2C).

### 3.2 ZCP improved serum and duodenum zinc levels in rat offspring born to zinc-deficient parental rats

**FIGURE 3:**
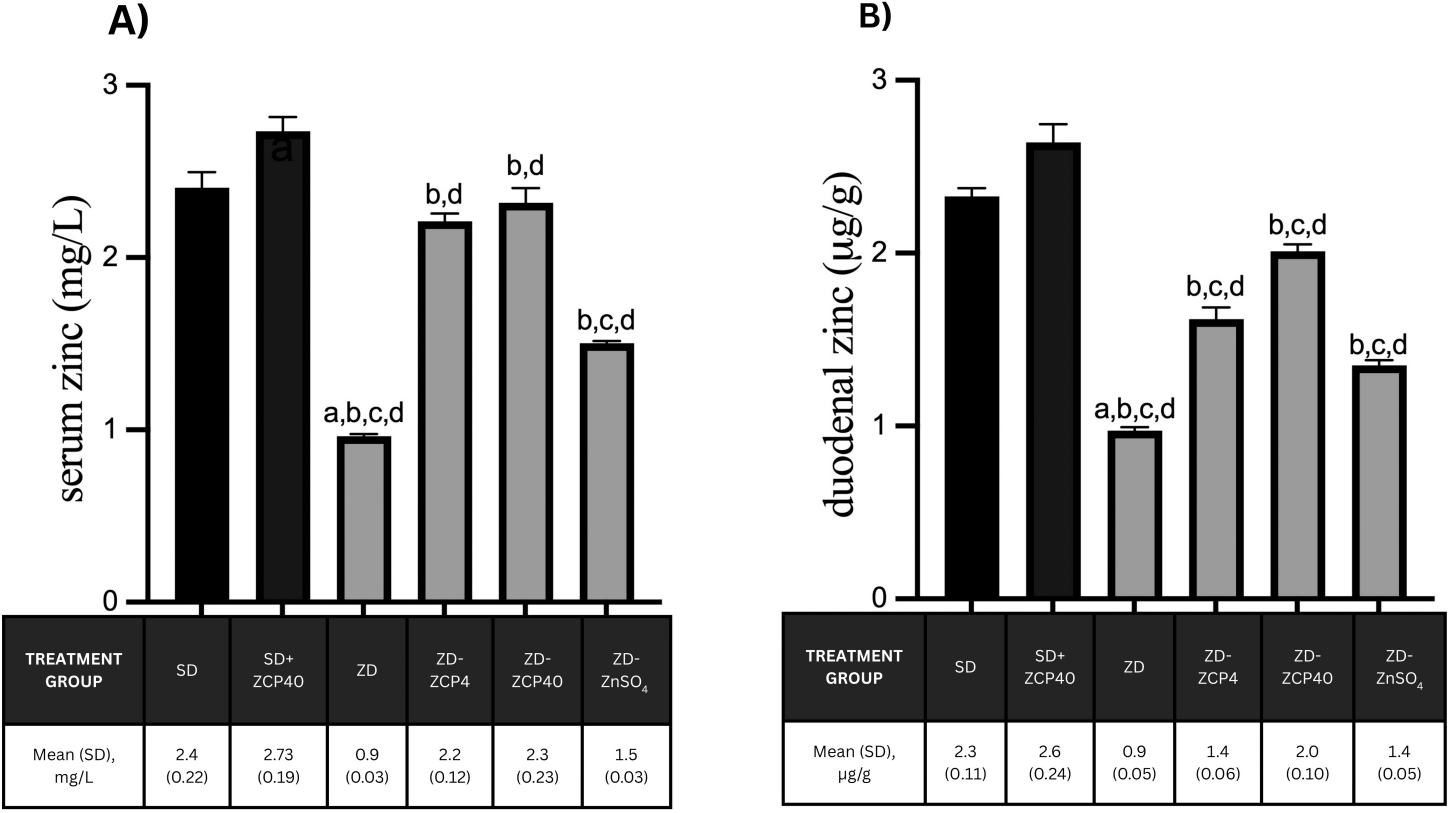
Zinc concentrations in the serum **(A)** and duodenum **(B)** of rat offspring fed either a standard diet or a zinc-deficient diet, treated with ZCP or ZnSO_4_. SD: standard diet + vehicle; SD + ZCP40: standard diet + ZCP 40 mg/kg BW; ZD: zinc-deficient + vehicle; ZD + ZCP4: zinc-deficient + ZCP 4 mg/kg BW; ZD + ZCP40; zinc-deficient + ZCP 40 mg/kg BW; ZD + ZnSO_4_: zinc-deficient + ZnSO_4_ 40 mg/kg BW. a: *p* < 0.05 vs SD; b: *p* < 0.05 vs ZD; c: *p* < 0.05 vs ZCP4; d: *p* < 0.05 vs ZCP40.

Treatment of zinc deficiency with both doses of ZCP (4 mg/kg BW and 40 mg/kg BW) has resulted in normalization of serum zinc concentrations and a significant increase in duodenal zinc concentrations. The increase in zinc concentrations after treatment with ZCP is significantly higher than that achieved with ZnSO_4_. Supplementation of ZCP to rat offspring in the standard diet caused a slight increase in serum and duodenal zinc concentrations (Figure 3).

### 3.3 ZCP restores body weight and hemoglobin levels in rat offspring born to zinc-deficient parental rats

**FIGURE 4:**
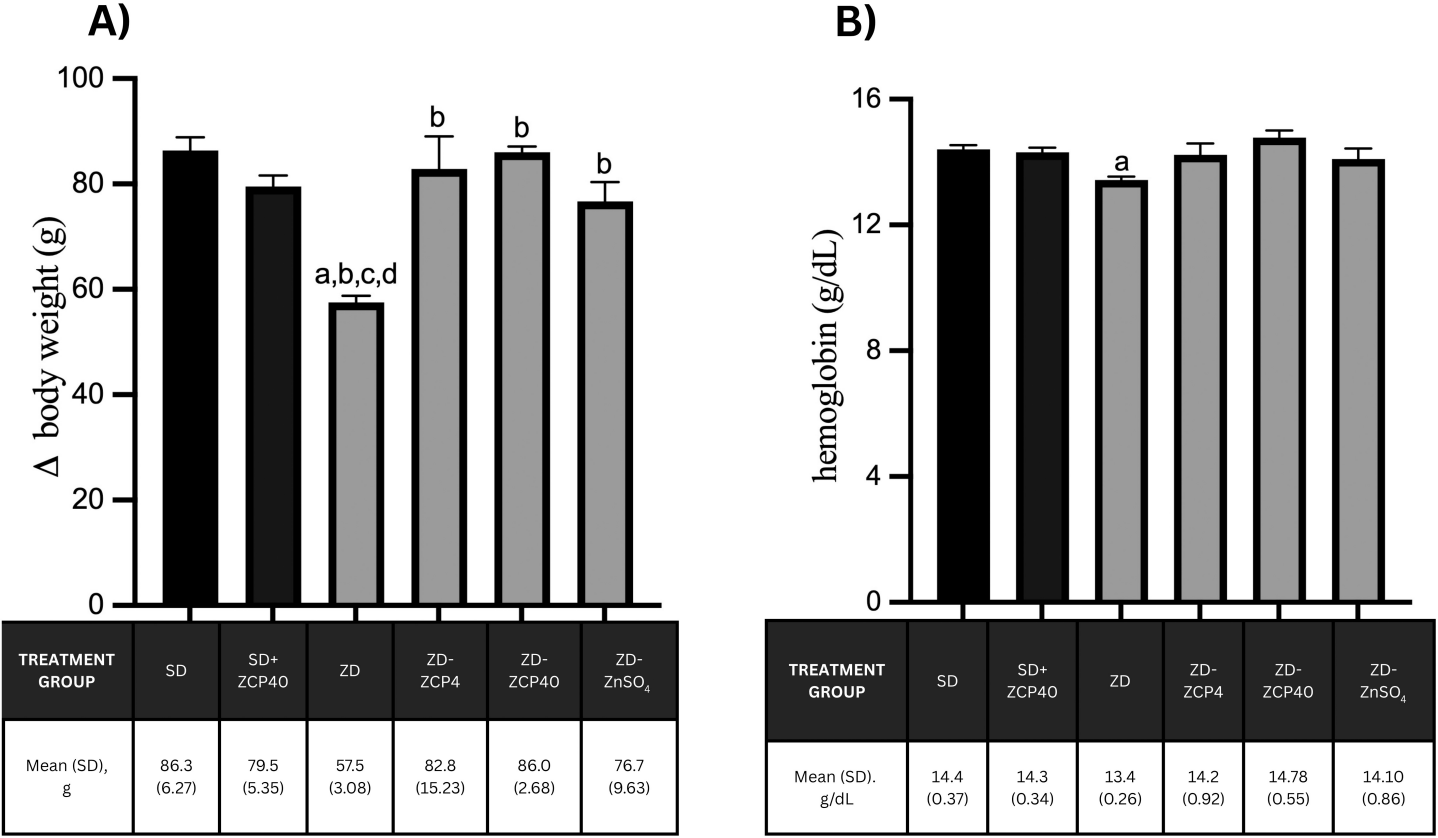
Difference in body weight before and after treatment **(A)**; and hemoglobin level **(B)** in rat offspring fed a standard diet and zinc-deficient diet, treated with ZCP or ZnSO_4_. SD: standard diet + vehicle; SD + ZCP40: standard diet + ZCP 40 mg/kg BW; ZD: zinc-deficient + vehicle; ZD + ZCP4: zinc-deficient + ZCP 4 mg/kg BW; ZD + ZCP40; zinc-deficient + ZCP 40 mg/kg BW; ZD + ZnSO_4_: zinc-deficient + ZnSO_4_ 40 mg/kg BW. a: *p* < 0.05 vs SD; b: *p* < 0.05 vs ZD; c: *p* < 0.05 vs ZCP4; d: *p* < 0.05 vs ZCP40.

Zinc deficiency led to a significant decrease in both body weight and hemoglobin in rat offspring. All active treatments, including ZCP at both doses and ZnSO_4_, restore body weight and hemoglobin levels in rat offspring with zinc deficiency (Figure 4). Supplementation of ZCP to the progeny in a standard diet does not cause any changes in body weight and hemoglobin of the offspring (Figure 4).

### 3.4 ZCP increases metallothionein levels and mRNA expressions of zinc transporters in rat offspring born from zinc-deficient parental rats

**FIGURE 5:**
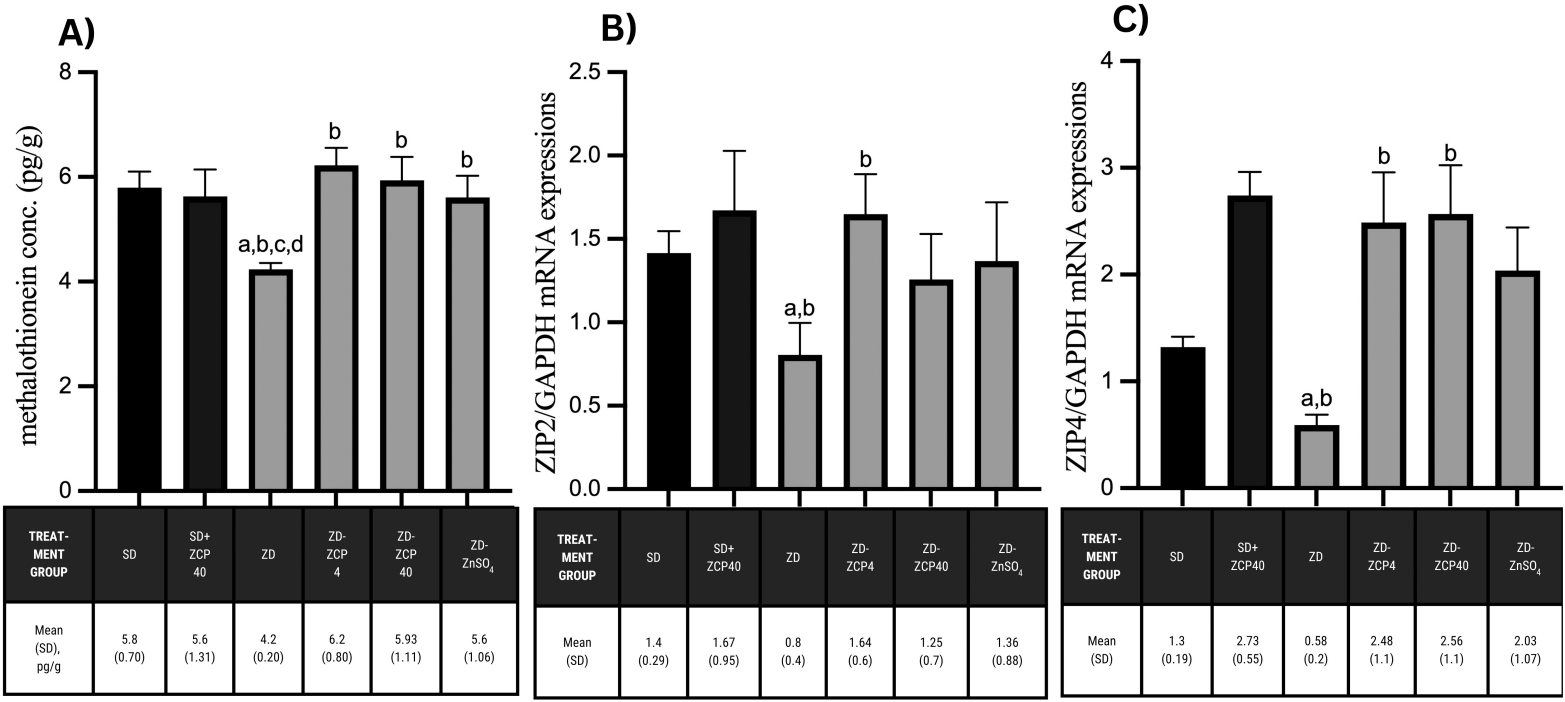
Metallothionein concentrations **(A)**; mRNA expressions of ZIP2/GAPDH **(B)**; and mRNA expressions of ZIP4/GAPDH **(C)** in the rat offspring subjected to a standard diet and zinc-deficient diet treated with ZCP or ZnSO_4_. SD: standard diet + vehicle; SD + ZCP40: standard diet + ZCP 40 mg/kg BW; ZD: zinc-deficient + vehicle; ZD + ZCP4: zinc-deficient + ZCP 4 mg/kg BW; ZD + ZCP40; zinc-deficient + ZCP 40 mg/kg BW; ZD + ZnSO_4_: zinc-deficient + ZnSO_4_ 40 mg/kg BW. a: *p* < 0.05 vs SD; b: *p* < 0.05 vs ZD; c: *p* < 0.05 vs ZCP4; d: *p* < 0.05 vs ZCP40.

Zinc deficiency has led to a significant decline in zinc metallothionein levels and reduced mRNA expressions of ZIP2 and ZIP4. The metallothionein concentrations of metallothionein were normalized after treatment with ZCP 4 mg/kg BW, 40 mg/kg BW, or ZnSO_4_. However, significant increases in the mRNA expression of ZIP2 and ZIP4 were observed after ZCP treatment at 4 mg/kg BW compared to the other two treatments. There were no changes in metallothionein concentrations or in the mRNA expressions of ZIP2 in the standard diet rat offspring groups; however, there was a slight increase in ZIP4 mRNA expression after treatment with ZCP (Figure 5).

## 4 Discussion

Zinc deficiency in children is shown to have impaired immune function, delayed development, and a higher risk of infectious disease. Zinc supplementation may enhance growth and development and lower the risk of illnesses in zinc-deficient children (7, 23, 24). The current approach to zinc supplementation for zinc deficiency includes both organic and inorganic forms. However, the efficacy of these treatments is limited by their low availability and gastrointestinal side effects such as nausea and vomiting (11). In the present study, we utilized zinc-chelating peptides from *Holothuria scabra* to improve zinc status in rat offspring born to zinc-deficient parents. Our results have demonstrated that the supplementation of ZCP from *Holothuria scabra* successfully normalizes the zinc serum concentrations as well as body weight in zinc-deficient rat offspring.

In our study, we successfully established a zinc-deficient animal model by feeding adult rats a low-zinc diet throughout adaptation, gestation, and lactation, resulting in reduced zinc levels in both parental rats and their offspring. In the present study, we quantified the zinc concentrations in the duodenum. While zinc is known to be absorbed throughout the small intestine, the highest absorption rate is reported either in the duodenum or the ileum (25, 26). At the same time, our previous findings indicate that the duodenum is the section where ZCP is optimally absorbed compared with the ileum and jejunum (21). However, it is essential to note that different segments of the other small intestine also play a role in zinc absorption.

Our study demonstrated that treatment with both doses of ZCP successfully normalized serum zinc levels and significantly increased duodenal zinc levels. ZnSO_4_, administered at the same dose as ZCP (40 mg/kg BW), was chosen based on previous studies on ZnSO_4_ supplementation in rats that used doses of 30–50 mg/kg BW (27, 28). Therefore, we selected 40 mg/kg BW as the standard dose for both ZnSO_4_ and ZCP. To test our hypothesis that ZCP has better absorption, we used a much smaller dose of ZCP (4 mg/kg BW), which our results confirmed; supplementation with ZCP at 4 mg/kg BW led to higher serum zinc concentrations compared to ZnSO_4_ at 40 mg/kg BW.

ZnSO_4_, though also improving zinc concentrations, cannot achieve the same level of improvement as ZCP. This is due to chelated zinc’s greater absorption efficiency compared to inorganic zinc (12, 25). Chelated zinc is bound to an amino acid, allowing it to be absorbed intact through the cell membrane into the systemic circulation, thereby bypassing competitive absorption pathways. This provides a direct, efficient route into the plasma that bypasses some of the rate-limiting steps of inorganic zinc absorption. On the other hand, inorganic zinc must be chelated with amino acids or other substances, such as a coenzyme, to be absorbed. In terms of inorganic zinc, the formation of complexes of ZnSO_4_ with phytates can reduce zinc absorption in the gut, leading to lower efficacy compared to chelated zinc (11, 25, 29).

In our model, zinc deficiency in the offspring also resulted in lower body weight, and supplementation with both doses of ZCP and ZnSO_4_ improved body weight. However, the improvement was not as pronounced as in offspring treated with ZCP alone. Zinc chelate is more effective at promoting weight gain in the offspring due to improved absorption and utilization (25). A study in cherry valley ducks showed that zinc glycine chelate exceeds ZnSO_4_ in increasing dietary intake and promoting body weight in young animals. The positive effects are linked to improved regulation of intestinal morphology, barrier integrity, and gut microbiota by zinc chelate compared to inorganic zinc (30).

Both zinc deficiency and zinc excess have been linked to contributing to anemia (31); in the present study, our zinc-deficient model resulted in a decrease in hemoglobin. Both types of zinc supplements, ZCP and ZnSO_4_, have a similar effect on improving the hemoglobin levels in zinc-deficient offspring. Zinc is an essential micronutrient that functions as a catalyst, structural component, and regulatory ion in various metabolic processes, including erythropoiesis. This study did not measure the interaction between iron and zinc; however, the hemoglobin levels examined offer an indirect reflection of this interaction. The presence of iron in hemoglobin affects enterocytes, which are related to intestinal permeability and, consequently, individual immunity (31). Human studies have demonstrated that zinc supplementation in patients with zinc deficiency and chronic anemia has led to resolution of anemia (31, 32).

To understand the role of ZCP in increasing zinc transport to the systemic circulation, we quantified metallothionein concentrations in duodenal segments of rat intestines. Metallothionein is a cellular peptide that binds minerals and plays a crucial role in zinc transport. Zinc bioavailability has been demonstrated to affect the synthesis of metallothionein (MT) within the body. Inadequate zinc absorption, due to its complexation with antinutrients such as phytate, has been shown to affect zinc availability in the body adversely. Both ZCP and inorganic zinc increase metallothionein levels (33, 34). Metallothionein binds to Zn^2+^ ions, the primary type of zinc required by the protein, which explains the similar effects resulting from ZCP and inorganic zinc. A zinc chelate is an organic form of zinc in which a ligand securely binds the zinc ion; nonetheless, this chelated zinc must be dissociated to liberate inorganic zinc (Zn^2+^) for binding with the cysteine residues in the metallothionein protein structure. Consequently, metallothionein does not directly associate with zinc chelates; it interacts with the inorganic zinc (Zn^2+^) released from them (33–35).

Metallothionein works collaboratively with zinc transporters (such as ZnTs and ZIPs) to regulate cellular zinc homeostasis. MTs act as buffers and stores for excess zinc. At the same time, zinc transporters facilitate the movement of zinc in and out of the cell and into intracellular compartments. The balance between these systems is crucial in preventing zinc toxicity and maintaining optimal cellular function (33, 34). Among the many zinc transporters, ZIP2 and ZIP4 have been identified as the primary transporters involved in zinc absorption (36–38). ZIP2 and ZIP4 are zinc influx transporters crucial for zinc homeostasis, with ZIP4 primarily responsible for dietary zinc absorption in the intestine and ZIP2 playing cell-specific roles. Their expression levels are linked to cellular zinc status, as demonstrated by age-dependent decreases in expression that correlate with lower intracellular zinc levels. ZIP4’s activity is also regulated by zinc-induced endocytosis, which reduces cellular uptake, while ZIP2 and ZIP4 can compensate for each other’s functions, especially during zinc deficiency (36, 39, 40).

Our study found that ZIP2 (SLC39A2) and ZIP4 (SLC39A4) zinc-deficient rat offspring had lower expression of ZIP2 and ZIP4 compared to offspring in standard diet groups. ZCP supplementation increases the expression of ZIP2 and ZIP4 in tissues, consistent with previous research showing that zinc transporter expression increases after zinc supplementation (41).

Our findings indicate that ZIP2 expression was markedly increased after administration of ZCP or ZnSO_4_, in response to the systemic zinc concentrations. The increased response suggests that ZIP2 may serve as an initial molecular sensor for zinc deficiency. Earlier *in vitro* studies on vascular endothelial and smooth muscle cells have shown that ZIP2 exhibits dynamic responsiveness to extracellular zinc, enabling it to achieve Zn homeostasis (42). Our observations corroborate this by providing *in vivo* evidence that peptide-bound zinc may specifically enhance ZIP2 expression, possibly through improved cellular uptake.

ZIP4, another leading zinc transporter in the small intestine (36, 43). Similar responses were observed after supplementation with ZCPs and ZnSO_4_ in the zinc-deficient offspring group. All active treatment groups have increased the mRNA expressions of ZIP4 transporters. While studies have shown that a more complex homeostatic mechanism regulates ZIP4 expression (43, 44), the response trend after zinc supplementation remains consistent with that of metallothionein and ZIP2. Thus, our data collectively showed that zinc supplementation in the form of ZCP and ZnSO_4_ affects zinc homeostasis, which includes metallothionein, ZIP2, and ZIP4.

The overall results of this study demonstrate that the lower dose of ZCP (4 mg/kg BW) produced an effect similar to that of the higher dose of ZCP (40 mg/kg BW) and to ZnSO_4_ (40 mg/kg BW). This difference may be explained by the greater solubility and stability of peptide-bound zinc (11), which facilitates absorption at lower concentrations. The transport of higher doses of ZCP via transporters may also explain the rate-limiting step for zinc entry into the cells.

The present study also confirmed that supplementing with ZCP in the zinc-sufficient group did not cause a significant increase in zinc levels or in any of the other variables studied, indicating a small risk of zinc toxicity. Data have shown that zinc toxicity from over-supplementation is rare but can lead to gastrointestinal adverse effects, including nausea, vomiting, or abdominal pain (45).

Our study has several limitations. The study was conducted over a relatively short period, which may have been inadequate for investigating long-term changes in zinc transporter expression. Secondly, transporter expression was assessed only at a single final time-point, which may limit the kinetic analysis of transporter regulation. We also did not analyze sex-specific differences in the offspring, which may not reveal the possibility of sex differences in zinc-chelate transport. Other than that, the use of a single species and reliance on surrogate biomarkers without functional outcomes should be considered in future efficacy studies.

Despite the limitation, our study confirmed that ZCP from *Holothuria scabra* is effective in treating zinc deficiency in the rat offspring. ZCPs, as third-generation zinc supplements, are designed to enhance zinc absorption. Our findings support this, as both ZCP doses (4 mg/kg BW and 40 mg/kg BW) significantly increased duodenal and serum zinc levels in zinc-deficient offspring, restoring them to near-normal levels. Finally, future studies should investigate the specific mechanisms of ZCP absorption, compare its long-term effects with other zinc supplements, and explore its impact on growth and outcomes in animal models.

## 5 Conclusion

In conclusion, ZCPs from *Holothuria scabra*, a third-generation zinc supplement, are superior to inorganic zinc (ZnSO_4_) in restoring zinc levels and normalizing body weight in zinc-deficient rat offspring. The effect of ZCPs at a dose of 4 mg/kg BW is similar to that of ZCP 40 mg/kg BW and greater than ZnSO_4_ 40 mg/kg BW. Therefore, the lower dose of ZCP is recommended for further development as the third generation of zinc supplements.

## Data Availability

Publicly available datasets were analyzed in this study. This data can be found here: s.brin.go.id/l/ZincPeptide_FrontierData.
